# Gender intentional approaches to enhance health social enterprises in Africa: a qualitative study of constraints and strategies

**DOI:** 10.1186/s12939-021-01427-0

**Published:** 2021-04-10

**Authors:** Kevin McKague, Sarah Harrison, Jenipher Musoke

**Affiliations:** 1 Cape Breton University, 1250 Grand Lake Road, Sydney, Nova Scotia Canada; 2 Gender Equality Specialist, Toronto, Canada; 3 BRAC Uganda, 880 Heritage Road, Nsambya, Kampala, Uganda

**Keywords:** Gender equality, Social enterprise, Community health workers, Africa, Uganda, Kenya

## Abstract

**Background:**

Health social enterprises are experimenting with community health worker (CHW) models that allow for various income-generating opportunities to motivate and incentivize CHWs. Although evidence shows that improving gender equality contributes to the achievement of health outcomes, gender-based constraints faced by CHWs working with social enterprises in Africa have not yet been empirically studied. This study is the first of its kind to address this important gap in knowledge.

**Methods:**

We conducted 36 key informant interviews and 21 focus group discussions between 2016 and 2019 (for a total of 175 individuals: 106 women and 69 men) with four health social enterprises in Uganda and Kenya and other related key stakeholders and domain experts. Interview and focus group transcripts were coded according to gender-based constraints and strategies for enhanced performance as well as key sites for intervention.

**Results:**

We found that CHW programs can be more gender responsive. We introduce the *Gender Integration Continuum for Health Social Enterprises* as a tool that can help guide gender equality efforts. Data revealed female CHWs face seven unique gender-based constraints (compared to male CHWs): 1) higher time burden and lack of economic empowerment; 2) risks to personal safety; 3) lack of career advancement and leadership opportunities; 4) lack of access to needed equipment, medicines and transport; 5) lack of access to capital; 6) lack of access to social support and networking opportunities; and 7) insufficient financial and non-financial incentives. Data also revealed four key areas of intervention: 1) the health social enterprise; 2) the CHW; 3) the CHW’s partner; and 4) the CHW’s patients. In each of the four areas, gender responsive strategies were identified to overcome constraints and contribute to improved gender equality and community health outcomes.

**Conclusions:**

This is the first study of its kind to identify the key gender-based constraints and gender responsive strategies for health social enterprises in Africa using CHWs. Findings can assist organizations working with CHWs in Africa (social enterprises, governments or non-governmental organizations) to develop gender responsive strategies that increase the gender and health outcomes while improving gender equality for CHWs, their families, and their communities.

**Supplementary Information:**

The online version contains supplementary material available at 10.1186/s12939-021-01427-0.

## Background

*“To achieve universal health coverage, explicit attention must be given to addressing inequalities, including interventions that counter unequal gender norms, prevent violence against women, and ensure women’s access to social and financial protection.”*

- Tedros Adhanom Ghebreyesus, Phumzile Mlambo-Ngcuka, and David Malone (2020: 1) [1]

There has been resurgence in interest in using community health workers (CHWs) to enhance frontline primary health care given their potential to fill gaps and reach remote and marginalized communities [2]. In light of this, health social enterprises are experimenting with models that allow for various income-generating opportunities to motivate and incentivize CHWs [3]. Health social enterprises are organizations with social objectives that generate revenue from their services and reinvest this surplus for the continued achievement of their social purposes [4]. Building on evidence that shows that improving gender equality contributes to the achievement of health outcomes [5], including maternal and child health (MCH) [6], health social enterprises utilizing CHWs in Africa have begun to consider the benefit of integrating gender equality to enhance CHW economic and personal outcomes [7].

When discussing gender equality, it is important to clarify the difference between sex and gender. Whereas sex refers to the biological or physiological characteristics of a person, gender refers to the “roles, behaviours, activities, and attributes that a given society may construct or consider appropriate for the categories of ‘men’ and ‘women’.” [8] The concept of gender equality, then, means that “women and men [and people of all genders^1^] enjoy the same status and have equal opportunity to realize their full human rights and potential to contribute to national, political, economic, social and cultural development, and to benefit from the results.” [9] Gender inequalities are largely well understood at the level of health and especially for MCH [10]. For example, many mothers still face limited negotiation power with partners and restricted autonomy in reproductive matters globally [11]. However, CHWs, particularly female ones, also face numerous gender-based constraints and inequalities that are generally less well understood or recognized within health systems [12].

## Gender equality as economic empowerment

At its root, gender equality is a fundamental human right for everyone [13], but research shows that gender equality also improves many development efforts [14] by enhancing economic development and productivity [15] and improving the health of populations around the world [5]. Sustainable Development Goal 5 aims to achieve gender equality globally and to empower all women and girls [16]. Research on women’s economic empowerment and gender equality, including a study by Acumen, examined how integrating gender can help to optimize social enterprise business models to improve both business and social impacts [17]. Acumen’s study demonstrated that social enterprises can gain tangible positive business impacts through better integration of women, including increased sales and profitability, and improved social impacts [17]. Acumen found that developing equitable systems also had positive personal impacts, suggesting an increase in employee satisfaction, retention, and increased innovation. However, the Acumen study did not consider the specific approaches of health social enterprises.

While the field of gender equality as an economic and business variable in global development settings is still new, initial data suggests that health social enterprises in Africa would benefit from supporting CHW’s overall economic empowerment through gender equality efforts, bringing about positive effects for CHWs themselves and community health outcomes [17].

## Community health workers

Community Health Workers (CHWs) are commonly used in resource-constrained and under-served settings, including many communities in Sub-Saharan Africa [18]. CHWs are generally chosen from the communities in which they live in order to help community members access basic primary health care [19]. They receive basic training according to various objectives, but it often includes prenatal and postnatal pregnancy care, the promotion of healthy behaviours (e.g., hygiene, immunizations, nutrition, family planning, sanitation, clean water), and the assessment and treatment of malaria and diarrhea [20].

It is estimated that 70% of CHWs globally are female [21]. They are often volunteers, although they also commonly receive some compensation for their activities, such as attending refresher training courses or participating in public health outreach campaigns [22].

## The pathway to gender intentional strategies

### Gender perspective

The first step in an organization’s movement toward gender intentional strategies and programs is gaining a gender perspective*,* which takes into account the gender-based differences of women, men, and people of all genders when looking at any action, policy, or program [23]. In short, a gender perspective equally considers and addresses the needs and interests of everyone, distinctly and explicitly. Merely shifting one’s perspective opens up the possibility of using specific tools to understand and use gathered information on gender issues for an organization and its context.

### Gender analysis

A primary tool for gaining a better gender perspective is a gender analysis. Gender analysis is a “systematic methodology for examining the differences in roles and norms for women and men, girls and boys [and people of all genders]; the different levels of power they hold; their differing needs, constraints, and opportunities; and the impact of these differences in their lives.” [5] A comprehensive gender analysis formalizes the intentions of having a gender perspective and deepens the underlying understanding of a gender. Importantly, effective gender analysis must also consider important interrelations and intersections with gender such as race, ethnicity, and age, for instance [24]. A gender analysis is important because if an organization does not fully understand and address key gender-based constraints, its CHWs can face unintended negative consequences and other issues such as increased risks to personal safety or high turnover.

For health social enterprises working with CHWs in Africa, a gender analysis is essential, as it aids in the integration of gender including the development of gender responsive and context-specific strategies designed to address critical gender-based constraints. It is important to note that gender-based constraints can vary significantly across health social enterprises depending on the country, social context, and business model [25]. Therefore, a robust and comprehensive gender analysis is vital to design appropriate gender equality strategies and interventions. A gender analysis should culminate in the development of a gender equality strategy or action plan that guides health social enterprises on their gender equality efforts.

### Gender intentionality

**Fig. 1:**
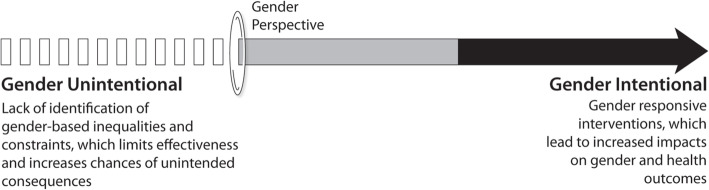
Gender Unintentional versus Intentional Efforts for Health Social Enterprises [27]

Gender analysis deepens the understanding of a health social enterprise on the influence of gender across their work, allowing their efforts to be more gender intentional. Gender intentionality means identifying and understanding gender inequalities, gender-based constraints, and inequitable norms and dynamics and taking steps to address them [26]. When an organization or initiative is unintentional in its efforts (via the lack of identification of gender inequalities and constraints), there is the potential for unintended or negative consequences, along with less effective results (Fig. 1).

### Gender integration

**Fig. 2:**
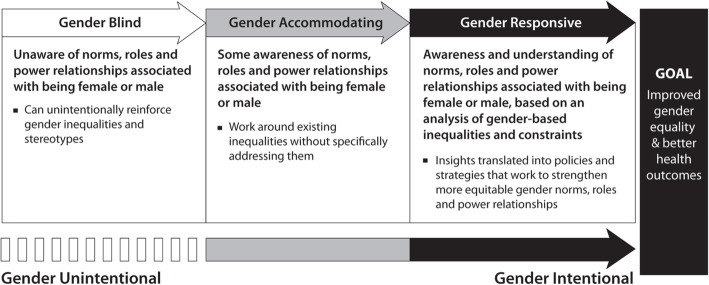
Gender Integration Continuum for Health Social Enterprises [27]

In order to consider gender intentionality further, the Gender Integration Continuum for Health Social Enterprises is a useful tool (Fig. 2). For health social enterprises, a strategy, intervention or action can be placed on the continuum to assess the degree of gender intentionality from being gender blind (on the left) to gender responsive (on the right).^2^ When a health social enterprise is operating gender blind, strategies, and interventions are unlikely to achieve their full potential and have the possibility to cause harm (even if unintentional) or to be exploitative.

From a gender equality perspective, health social enterprises should strive to move along the continuum of gender intentionality. If a health social enterprise is working around existing gender inequalities but they are doing so without critical examination (via gender analysis) of the influence of gender as it relates to their work, then they can be said to be gender accommodating. Being fully gender intentional also involves explicitly addressing gender imbalances.

#### Becoming gender responsive

From a gender equality perspective, the aim of a health social enterprise’s gender efforts is to be gender responsive. Gender responsive programs seek to understand, respond, and transform gender relations to promote equality and achieve program objectives. Gender responsive approaches use gender analysis to systematically understand and examine the influence of gender roles, norms, and dynamics. These approaches aim to change inequitable norms and dynamics or to strengthen already equitable systems for improved gender equality.

For instance, related to health, gender responsive approaches seek to “change gender norms that restrict women and men’s access to health services and realization of good health. They question and challenge the unequal distribution of power, lack of resources, limited opportunities and benefits, and restrictions on human rights.” [5] Examples of gender responsive strategies may include supporting changes in gender roles, greater equality in the distribution of goods and services, sharing power at home, and increasing men’s engagement in women’s health.

It is important to note that working with women is not the same as integrating gender or being gender intentional. For instance, considering women as they relate to maternal health would consider the issues that women face uniquely, such as reproductive rights. Albeit important, being gender intentional goes beyond this consideration and analyzes the influence of gender including sociocultural norms and power relations as they impact maternal health outcomes. Gender responsive approaches understand that women and men may face different gender-based constraints, risks, and health outcomes and that women and men’s health outcomes are interconnected.

Although the importance of understanding gender dynamics and constraints in achieving greater health outcomes and gender equality is increasingly recognized, and the efforts of health social enterprises working with CHWs in Africa is growing significantly, no empirical research has yet been undertaken at the intersection of these two significant trends. This study was designed to address this gap in our knowledge.

## Methods

### Study area

Gender norms can vary depending on many factors including cultural norms, ethnic groupings, the laws and history of a country, whether primarily a rural or urban setting, and socio-economic status. Generally speaking, gender norms in Uganda and Kenya can be characterized as patriarchal, where men are typically are viewed as the head of the household, are the primary property owners, hold positions of power and social privilege, and predominate in political offices and other decision-making roles in society [28].

The study was conducted in Kenya and Uganda; two low-to-middle income countries located in East Africa. The population in these sub-Saharan African countries is relatively young, with approximately 65 and 77% of Kenya’s and Uganda’s population being 25 years of age or younger, respectively [29, 30]. The majority of the population cannot afford to pay for health care, the poor are less likely to utilize health services when they are ill, and wide disparities in utilization exist between geographical regions and between urban and rural areas [31]. Many individuals and households in the region experience social and health inequalities in relation to accessing basic services such as health care, water and sanitation, food, and decent housing [29, 30]. Access to primary healthcare services is a challenge either because of distance to facilities or economic barriers. In spite of efforts to increase access to health services at the community level, wide disparities in utilization exist between geographical regions and between urban and rural areas with urban areas having more access to basic services than rural areas [31]. In Uganda for example, the rural-urban divide is reflected in the maternal and newborn health indicators where coverage of skilled attendance at birth is 52% in rural areas, compared to 89% in urban areas [32].

In addressing these health gaps, the government of Uganda through the Ministries of Health introduced a community strategy that emphasized the need for CHWs (locally referred to as Village Health Teams) not only in rural areas but also in urban centres [33]. The Kenyan government also introduced CHWs into the healthcare system with the goal of making primary healthcare services accessible to all, specifically those in underserved communities [34, 35].

For this study, we drew our participants from CHWs that were involved with four health social enterprises in Nairobi county (Kenya), Kampala, Masaka, and Bukomansimbi districts (Uganda). In Nairobi, the participants were CHWs working in an urban informal settlement where healthcare provisioning is extremely limited, poorly resourced, and difficult to access, making the extended reach of CHWs important. Over 35% of Kenyans are currently living in urban areas, and 75% of people in urban areas live in informal settlements [29, 34]. These communities are characterised by high levels of poverty, insecurity, and inadequate access to basic social services and amenities. In Uganda, Kampala district was selected to represent the urban population with similar characteristics. Masaka and Bukomansimbi districts of Uganda represented rural communities where food crop agriculture is the main economic activity. Masaka has a rich cultural heritage with diversity of ethnicities of about 40 ethnic groups, although the majority are Baganda, and most of the tribes practice the Baganda culture [30].

### Study organizations

To understand how health social enterprises in Africa can contribute to greater health outcomes and gender equality by addressing gender-based constraints for CHWs, we designed a study to answer the following research question: *What are the key gender-based constraints and strategies for CHWs working with social enterprises in Africa and where are they found?*

To answer this question, we designed an in-depth qualitative study that would conduct key informant interviews and focus groups with four health social enterprises in Africa using CHWs. Qualitative studies are suitable for understanding social issues including gender norms and related constraints and opportunities [36]. We primarily focused on the organizational level of analysis (the health social enterprise), however our qualitative approach also allowed us to consider the individual level of analysis of CHWs and the social and economic context in which the CHWs and health social enterprises were embedded. Our qualitative approach was problem-driven, and oriented to explaining phenomena in a complex social environment [37]. Our research approach was primarily inductive, especially during the earlier phases of data gathering and analysis, but also included deduction as we consulted the literature on gender issues and CHWs.

To understand gender issues and constraints in the work of CHWs working with health social enterprises, we selected four organizations for our study, which were sampled to cover diversity in terms of countries of operation, whether they worked primarily in rural or urban areas, whether CHWs operated primarily door-to-door or were based in a clinic, and whether organizations worked with only women or worked with male and female CHWs (see Table 1). The four organizations selected were BRAC Uganda, Access Afya, Healthy Entrepreneurs, and LifeNet International.

**Table 1:** Sampled Health Social Enterprises in Africa working with CHWs

	BRAC Uganda	Healthy Entrepreneurs	Access Afya	LifeNet International
Number of CHWs	4000 in Uganda	4000	100	100
Countries of Operation	Uganda, Tanzania, Sierra Leone, Liberia, South Sudan	Uganda, Tanzania, Kenya, Ghana	Kenya	Uganda, Democratic Republic of the Congo, Burundi, Malawi
Geographic Coverage	Rural and urban	Rural	Urban slums	Primarily rural
CHW operation model	Door-to-door	Door-to-door	Clinic-based	Clinic-based
Gender	Women only	Women and men	Women and men	Women and men

We began by reviewing the research literature [3] as well as the leading gender analysis frameworks and tools [38]. We then conducted 36 key informant interviews and 21 focus group discussions in Uganda and Kenya (for a total of 175 individuals: 106 women and 69 men). Data were collected between March 2016 and May 2019 which allowed for sufficient time to become familiar with the organizations, the health systems, and the gender norms in the regions of Uganda and Kenya where they operated. Data were collected in Kampala, Nkoni, and Bukomansimbi in Uganda and Nairobi in Kenya. The timeline, locations, and types of data collected is illustrated in Table 2.

**Table 2:** Data Collection Timeline

	2016 March, April	2017 March, May	2018 March, May			2019 May				
	Uganda	Uganda	Kenya	Uganda		Kenya	Uganda			
	Kampala	Kampala	Nairobi	Masaka	Kampala	Nairobi	Nkoni	Bukomansimbi	Masaka	Kampala
Location	Urban slum	Urban slum	Urban slum	Rural	Urban slum	Urban slum	Rural	Rural	Rural	Urban slum
Interviews	**6** – 1 female – 5 males	**8** – 3 females – 5 males	**7** – 7 females		**4** – 3 females – 1 male	**5** – 4 females – 1 male				**6** – 4 females – 2 males
Focus Groups	**1** – 5 females	**3** – 5 female CHWs – 5 male partners – 5 female patients	**4** – 7 female CHWs – 4 male CHWs – 4 male partners – 6 female patients	**3** – 11 female CHWs – 11 male partners – 7 female patients		**2** – 6 female CHWs – 4 male partners	**3** – 12 female CHWs – 6 male CHWs – 6 male partners	**2** – 9 female CHWs – 7 male partners	**3** − 5 female CHWs – 5 male partners – 5 female patients	

### Individual interviews

Interview data were collected face-to-face during in-person visits attended by at least one coauthor, although typically all three coauthors were present. Interviewees were purposively selected from the four organizations we studied (including founders, executive directors, CHW supervisors, front line workers, and senior leaders). Additional expert interviews were carried out with practitioners, researchers, and experts from other local organizations knowledgeable about gender issues and community health workers. To elicit the most valuable interview data, we struck a balance between a structured process with predetermined interview questions and embracing deviations initiated by the interviewees on topics relevant to our exploration. We made every effort to be fully present with interviewees, attentive to verbal and non-verbal cues, and sensitive to cultural norms during interviews and focus groups. We took efforts to establish a relaxed and comfortable setting for our interviewees to encourage openness and reflection [39]. Interviews ranged from 30 to 90 min.

### Focus group discussions

For the focus group discussions, seven were with female CHWs, two with male CHWs, seven with the male partners of female CHWs, and five with patients of CHWs (mixed gender). During this time, the coauthors also observed participants social interactions including relationships and dynamics between men and women. As the study progressed, groups were focused on filling remaining gaps in our understanding. Focus groups were conducted either directly in English or through a translator in a local language. All qualitative data were recorded, transcribed, and coded in light of the research question [40]. An example focus group and interview discussion guide is included in Additional file 1 Annex 1. Focus groups ranged from one to 2½ hours.

### Data analysis

In analyzing the data from interviews and focus groups we also stayed open to understanding the impacts of gender on other non-CHW employees, such as clinical assistants, clinical officers, and managers. Data was gathered and analyzed iteratively as the study progressed and we triangulated between interviews, focus groups, and the literature to enhance the reliability and trustworthiness of the findings [41]. The systematic use of iteration and triangulation was aimed at discovering the social norms related to gender and related constraints that CHWs faced, and strategies suggested to address them.

As interview and focus group data were collected, we began coding for gender-related constraints that CHWs faced, strategies to overcome the constraints and the conceptual ‘location’ where the issues were situated. We used both deductive and inductive coding. Our deductive coding started with some predefined codes related to our research question. These codes included: gender-based constraint and strategy to overcome constraint. We used inductive coding to understand the ‘locations’ of where we were finding gender issues (e.g. between the CHW and her partner, etc.) as well as to categorize gender-based constraints that CHWs faced and strategies to address them. Codes were weighted and organized hierarchically based on the perceived importance assigned to the particular gender issue at hand. Perceived importance and the structuring of the codes and their underlying themes was determined through discussions between coauthors.

Analysis and collection proceeded iteratively as data was gathered over the 3 years of the study. As we progressed, our themes and categories became progressively clearer until our framework and findings became finalized. During the data collection and analysis process the emerging themes and categories were cross-checked between interview data, focus group data, and related findings in the literature. The multiple sources of data allowed for triangulation and corroboration which served to enhance the validity of the findings. We presented early versions of our emerging framework and findings to key interviewees to guard against premature conclusions as a result of human information-processing biases [42].

The coding of our data into constructs and a framework was done by hand. We aimed to code at various levels of abstraction, while remaining grounded in the data. We split or consolidated the lists of constraints and strategies and the constructs that comprised the framework until further data gathering and analysis continued to fit our models without need for further refinement. To maximize the reliability of our categories, constructs and framework, the analysis and coding process included ongoing conversations between authors and the construction of diagrams and tables to organize and represent the data [43].

During the process of coming to the final version of our framework and categories of constraints and strategies we searched for what we believed was, on balance, the findings that best fit the data [44]. We continued our refinement of our framework and categories until the model and lists became stabilized and new data and analysis no longer resulted in refinements or changes.

## Results

### The gender integration framework for health social enterprises

The first findings to emerge from the analysis of the data was the answer to the question *‘where are gender-based constraints and strategies found?’* The data revealed four significant areas where gender-based constraints and strategies could be found:

1. within the health social enterprise itself
2. between the social enterprise and CHWs
3. between CHWs and their domestic partner^3^
4. and between CHWs and their patients

The data identified that identifying and addressing gender-based constraints in these four key areas offered the greatest potential to contribute to improved gender equality and health outcomes for all actors. The answer to the question, ‘*What are the key gender-based constraints and strategies’* were found to correspond to these four areas. Our data revealed that greater health outcomes and gender equality could be achieved through four interrelated pathways (corresponding to each of the four areas):

1. Equitable policies and systems;
2. Gender responsive training, support, and incentives;
3. Appropriate partner engagement; and
4. Gender responsive design and marketing of MCH products and services.

**Fig. 3:**
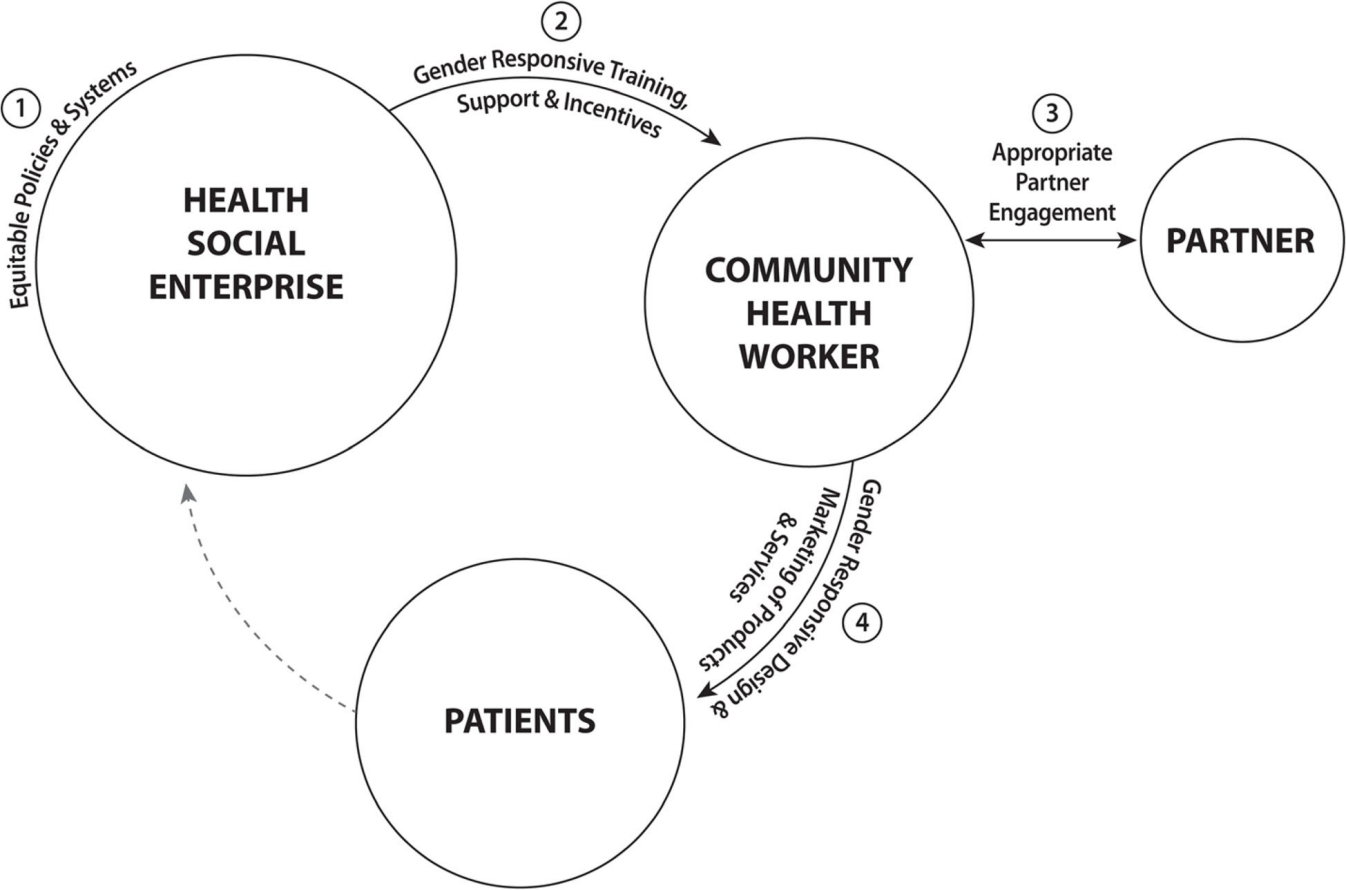
Gender Integration Framework for Health Social Enterprises

Figure 3, the *Gender Integration Framework for Health Social Enterprises*, illustrates these four key areas and their four interrelated pathways.

Further data analysis revealed the possible gender-based constraints with each of these four pathways. The specific constraints and their corresponding strategies are described further below. Our research identified seven primary gender-based constraints for female CHWs:

1. High time burden and lack of economic empowerment
2. Risks to personal safety
3. Lack of career advancement and leadership opportunities
4. Lack of access to needed equipment, medicines and transport
5. Lack of access to capital
6. Lack of access to social support and networking opportunities
7. Insufficient financial and non-financial incentives

We mapped the gender-based constraints and strategies on to the four pathways, with the results presented below.

### Equitable policies and systems

An early step in our data gathering and analysis was to understand how gender influences the policies and systems in a health social enterprise (part of the first pathway identified). Each organization, in its own way, had an understanding of the effects of gender and sociocultural norms, dynamics, power relations, and gender-based constraints that affected the CHWs they worked with, as well as various strategies to address them. The gender-based constraints that CHWs, particularly female CHWs, are likely to face and the potential gender responsive organizational policies and systems to improve gender integration are further described below.

#### High time burden and lack of economic empowerment

Female CHWs face many demands on their time and have to balance multiple roles, responsibilities, and income-generating activities. For example, many CHWs are farming and running a business such as selling fruit, vegetables, food products or tailoring in addition to their CHW work. Many CHWs work late into the evening (10 pm to midnight) or early in the morning (4 am to 6 am) to facilitate these demands. Female CHWs shared that balancing their roles was particularly difficult because the household burden is solely on their shoulders due to gender and sociocultural norms. As a female CHW from Nkoni stated, “We have the issue of clients coming late in the evening when you should be making food for your children and yet they need service. In some cases, you serve one child, then another and another and you end up feeling that you are taking all your time to really get the patients taken care of. It is draining.” Since most CHWs are volunteers, the time spent on CHW work (and the overall expectations) is high relative to the overall financial remuneration that they receive. Workplace actions and policies to address the high time burden and a lack of economic empowerment included: ensuring appropriate remuneration for CHWs time; developing opportunities for income generation, such as selling health products; and defining policies that support CHW work–life balance, including clear job descriptions with number of hours of expected work per week.

#### Risks to personal safety

The work of a CHW often requires women to travel alone and/or at night, especially in the case of labour and delivery or health emergencies. Personal safety was a primary concern raised by many CHWs.

Workplace actions and policies to promote CHW safety included: training; encouraging support from male partners when they need to accompany female CHWs to attend to a patient at night; and partner male and female CHWs to work together, if context appropriate.

#### Lack of leadership and career advancement opportunities

Female CHWs often face limited opportunities to advance their careers and take additional leadership roles due to levels of education and training and restrictive sociocultural norms. Female CHWs also shared that they have limited opportunities to connect with each other or other leaders. Workplace actions and policies to advance careers included: facilitating opportunities for training and capacity building; providing clear pathways for career advancement, promotion, and leadership; and providing mentoring or coaching opportunities between and among CHWs, with an emphasis on supporting female-to-female connections.

### Gender responsive training, support, and incentives

When analyzing data related to the second pathway, gender responsive training, support, and incentives, we found that CHWs, particularly female CHWs, often had limited education, literacy, and learning opportunities. They also often lacked access to needed equipment and medicines, transportation, and capital, as well as social supports and sufficient financial and non-financial incentives. As a CHW in Bukomansinbi stated, “I am requesting for refresher trainings just to give us the right information that we can give to our clients.” To address these gender-based constraints, we found that health social enterprises could look to provide training and professional development opportunities, and the appropriate tools, resources, and supports for CHWs to perform their roles effectively. They could also facilitate access to transportation, financial services, social support and networking opportunities, and ensure that there are adequate financial and non-financial incentives.

In order to increase gender intentionality, we found that it was important that all training, support, and incentives be targeted to the specific needs and priorities of both female and male CHWs, otherwise health social enterprises risked less effective implementation and other potential negative side effects, such as discontented CHWs and high turnover.

#### Limited education and learning opportunities

Generally, CHWs are selected from the community in which they live. Often CHWs, particularly female CHWs, have only basic levels of education, literacy, and training. As a female CHW in Nkoni stated, “We feel like we need more training for us to be able to provide efficient care to the community.” In addition, CHWs often struggled to deal with difficult or aggressive patients, especially pregnant single mothers. “We only have treatments for the kids but when mothers come, they bring children who are over 5 years and they insist on being treated. If you deny them the treatment, they go cursing you (Female CHW, Nkoni). CHWs also often lacked skills in business and financial literacy, which limited their economic empowerment and autonomy. When CHWs did not have adequate education or training, we found they were unable to provide services effectively and further build their knowledge, skills or confidence. Actions to improve education and learning included: regular refresher training; communication skills training and basic literacy; conflict management and negotiation training; business and financial literacy training; and empowerment training, including agency, leadership and decision-making training.

#### Lack of access to needed equipment and medicines

Proper access to needed equipment and supplies is needed by CHWs in order to do their job effectively, but proper equipment and medicines also assist CHWs in being perceived as legitimate health providers in the eyes of community members. As a female CHW from Nkoni stated, “The drug supply is a challenge in terms of drug stock running out. Some cases we do not have the drugs and the people come they need the care, but we do not have the drugs.” Actions to ensure access to needed equipment, medicines, and supplies included: managing supply chains to reduce stock-outs: considering the importance of t-shirts and other identifiers of a CHW’s role and affiliation as symbols of legitimacy by CHWs and community members; ensuring CHWs have access to needed equipment, such as a bag for carrying medicines, a thermometer, and gloves. In some contexts, gear for the rainy season (i.e. umbrellas, boots, and raincoats) were also important for going door-to-door to visit patients (as a female CHW from Bukomansimbi stated, “There is need to provide rainboots, courts, umbrellas to make our work quite easy during the wet seasons”); and providing greater access to a work mobile phone or tablet for both personal and work use.

#### Lack of transportation

CHWs, especially female CHWs, cited transport as one of their primary challenges. CHW work often requires transportation to home visits, especially if a community is geographically dispersed and patients need to attend a clinic (as when a patient is in labour). “We have the challenge of transportation within the villages which are far apart” (Female CHW, Nkoni). Transportation is significantly influenced by gender, as women often have limited mobility and a lack of funds to pay. As a female CHW in Nkoni stated, “Men find it easy to get transport unlike the women who are unable to cycle.” Actions to increase access to transportation included: providing transportation allowances for CHWs and facilitating access to context-relevant modes of transportation, such as female-friendly bicycles or motorcycles, where applicable.

#### Limited access to capital

For CHWs who generate some income from selling medicines and other health products, the ability to purchase inventories of medicines is important to be able to both serve the needs of their patients and generate income. CHWs, particularly female CHWs, are usually living in remote and rural areas with low income and face significant capital constraints compared to men. Actions to facilitate access to capital included: support opportunities for CHWs to save; support CHWs to have access to loans to purchase medicines; pay CHWs a salary or provide greater allowances for attending events, such as refresher trainings; and facilitate income-generation opportunities.

#### Lack of social support or networks

CHWs shared that they often feel isolated in their roles. Despite their important work, CHWs are often situated “outside” of the health systems they work with. They expressed having limited opportunity to connect with others, even other CHWs working within the same health social enterprise. Actions to facilitate access to social supports and networks included: supporting relationship and network development through interactions among CHWs, including regular meetings and refresher training; and providing mentoring or coaching opportunities among CHWs, with an emphasis on supporting female-to-female connections.

#### Insufficient financial and non-financial incentives

One of the main motivators for CHWs is an interest to help and serve their community. Additionally, CHWs appreciate the status, respect, visibility, and connections that typically come with the role. CHWs also value the greater level of health knowledge that they attain through their CHW training and access to medicines and health products for their families. Actions to ensure sufficient financial and non-financial incentives included: providing more opportunities for income generation, such as training in business and financial literacy; facilitating access to loans and savings; ensuring access to an adequate basket of medicines and health products to sell; providing appropriate remuneration; ensuring appropriate stipends and compensation for training, refresher training, and monthly meetings; supporting the ability of CHWs to help others in the community through good quality training and supervision; and supporting CHW, particularly female CHW, status and respect in the community. As a female CHW in Bukomansimbi stated, “If we could have some transport refunds to attend the cluster meetings that will be quite helpful to us.”

### Appropriate partner engagement

The third pathway, appropriate partner engagement, was found to be an important factor for many CHWs where the partner can be an impediment to or enabler of CHW’s success. We found that if an organization understood this important gender-based dynamic, they could take strategic actions to encourage partner support for CHWs, such as explicitly defining the partner’s role and increasing communication, awareness, and appropriate engagement as a CHW supporter. We found that although there has historically been a lot of emphasis on the importance of engaging men and fathers through MCH initiatives, little to no attention was paid to the partners of CHWs. The relationship between the CHW and their partner was found to be an essential gender dynamic to recognize, understand, and support.

We also found that the CHW’s partners can be a critical (but often invisible) enabler of success. As a female CHW from Nkoni stated, “Sometimes our husbands help in terms of transports and in collecting the drugs for us. It is quite important to us. The men are very key in even taking up the role as a [CHW]. If they are against it then you will have to come out of the role.” Frequently, female CHWs were being supported by their male partners in various ways, including providing money for transport or purchasing medicines, accompanying the CHW on night visits for safety, and communicating with community members who come to the CHW’s home when she is out. As a male partner of a CHW from Bukomansimbi stated, “I often help if a client comes and she is not around, I call to let her know and just keep the patient comfortable. I also give her a ride to the centres to collect the drugs and when she is going to do home visits.” Given that many CHWs are volunteers, often in severely impoverished communities, this kind of support was often overlooked.

However, in contexts where the CHW’s partner maintained strong control and decision-making power at the household level and CHWs did not have strong agency, partners could be an important limiting factor over a CHW’s ultimate effectiveness. A female CHW from Bukomansimbi stated: “To me, marital issues are so hard to really deal with. Some of the men feel that we are making some earnings and we should now take care of the family needs. In light of this dynamic, we found a health social enterprise could consider what gender responsive strategies would be most effective in enhancing partner engagement. Actions to maximize partner support and minimize partner resistance included: explicitly defining the partner’s role. Partners (and CHWs themselves) shared that it would be helpful for partners to improve their support if health social enterprises would communicate their role to partners and recognize the importance of the partner’s support and involvement; and improve partner awareness, education, support, and engagement. As a male partner of a CHW from Bukomansimbi stated, “I wish information could be passed to other people in this community on the benefits of being a CHW and how beneficial it is for the community so that we have more people who are actively involved in being a CHW.” As a different male partner of a CHW from Bukomansimbi stated, “We are left out.”

### Gender responsive design and marketing of MCH products and services

In the fourth pathway, gender responsive design and marketing of MCH products and services, an analysis of the data revealed gender-based constraints related to family planning and gender-based violence. The need for women to have autonomy and greater decision-making power over their reproductive health and the need for women to be free from risk of domestic violence is central to gender equality.

#### Family planning

Family planning was an important issue for health social enterprises with respect to design and marketing of MCH products and services. Family planning is also highly influenced by gender and sociocultural norms. Patients of CHWs shared that the decisions around family planning (for instance, how many children to have and when) was not theirs to make (or theirs to make alone). Actions to ensure gender responsive family planning included: segmenting female patients and not considering them as one homogenous group. It was important to recognize that different women may have varying needs and face different gender-based constraints. For instance, the needs of a young, single pregnant mother may be very different from an older, married women with multiple children. Other actions to ensure gender responsive family planning included: educating and counselling couples together (rather than individually) and providing counselling to partners (as well as female patients). Additionally, some social enterprises used male and female CHW pairings so that man-to-man and woman-to-woman discussions and counselling can occur.

#### Gender-based violence

Gender-based violence was found to be an important issue for CHWs and their patients. There are many ways and degrees to which gender-based violence manifests. For example, some female CHWs may be at increased risk as their roles change (both within and outside the household) or as they become more economically empowered. CHWs can struggle to provide services when men in the community do not have work. Whatever the situation may be, it is important for health social enterprises to strive to understand how this issue may or may not be impacting their work. To address the issue of gender-based violence with female patients, health social enterprises indicated that they can: engage and educate patients and partners; consider male and female CHW pairings in order to support female patients and their partners; and ensure CHWs have information for referrals to counselling or other support services and organizations. As a male CHW from Nkoni stated, “We also have cases whereas the men, we have women who prefer to talk to a man rather than a woman. So, in such cases like providing family planning services, we come in to provide the service, so we share some responsibilities [with the female CHWs].”

## Discussion

There has been a resurgence in interest in using CHWs to enhance frontline primary health care given their potential to fill gaps and reach remote communities. In light of this, health social enterprises (as well as governments and non-governmental organizations) are experimenting with CHW models that allow for various income-generating opportunities to motivate and incentivize CHWs. However, evidence shows that improving gender equality contributes to the achievement of health outcomes by CHWs, although this remains under-studied [45]. The current research addresses this gap.

Across the world, progress on gender equality has been uneven, “with those facing multiple forms of discrimination (e.g. on the basis of ethnicity, class, disability) and deprivation being those most likely to be left behind.” [1] Moreover, the Covid-19 pandemic has disproportionately affected women, who constitute the majority of frontline workers and CHWs worldwide [1]. The pandemic has seen increased reporting of domestic violence, heightened economic insecurity, and the disruption of protective social networks and access to sexual and reproductive health services [1]. Investing in removing obstacles to female CHWs’ work and improving gender equality can save lives, improve community health, and protect human rights.

In order to improve both gender equality and health outcomes, we found that it is important that health social enterprises understand the gender-based constraints that CHWs face, especially female CHWs, so that they can provide community health services more effectively. We found that addressing gender-based constraints has the added benefit of further empowering CHWs thereby increasing the social benefits for themselves and their families. The findings of this study offer some guidance for health social enterprises working with CHWs in Africa to further enable their CHWs to be more effective and empowered.

A health social enterprise’s response to gender issues exist along a continuum of gender intentionality. A health social enterprise’s actions can range from being gender unintentional to intentional. On the unintentional side, being gender blind means risking negative unintended consequences for both CHWs and the business, reinforcing inequitable gender and sociocultural norms, and limiting overall effectiveness. On the intentional side, being gender responsive means systematically understanding norms, roles, and power, along with key gender-based constraints (via gender analysis) and working to either reduce inequalities or inequitable norms or to strengthen existing equitable systems. Being gender responsive also requires conducting ongoing gender analysis and responding to gender-based constraints for both women and men (and people of all genders) as they emerge; it means working to overcome gender-based constraints by employing innovative strategies and solutions for improved gender equality.

Our research found seven primary constraints for female CHWs: high time burden and lack of economic empowerment; risks to personal safety; lack of career advancement and leadership opportunities; lack of access to needed equipment, medicines and transport; lack of access to capital; lack of access to social support and networking opportunities; and insufficient financial and non-financial incentives. Our research also found that a health social enterprise can explore gender issues through four interrelated pathways: equitable policies and systems; gender responsive training, support, and incentives; appropriate partner engagement; and gender responsive design and marketing of MCH products and services. Specific strategies emerged to help CHWs and health social enterprises be more gender responsive in addressing constraints in each of these four areas.

Although there is increasing recognition of the need to engage male partners to promote gender equality [46], little empirical research has focused on male partners’ influence on female CHWs ability to perform their roles. Where research has been done, the focus has typically been on the “content” of messages aimed to raise awareness among men [47]. The findings from this study revealed insights on the importance of defining the partners’ role in CHW work including recognizing the support that male partners can provide to support CHW’s work.

Social enterprises have been considered as having enhanced potential for improving gender equality because social enterprises are oriented toward addressing social issues more broadly and are often aligned with the values of equality and inclusion specifically [48]. However, existing research has tended to focus on women-focused projects by social enterprises and providing products and services to female customers, and less on identifying gender-based constraints and solutions for women as employees or volunteers (such as CHWs). No other study to our knowledge has considered gender issues for CHWs working with health social enterprises.

Health social enterprises operate in many different markets with dissimilar economic and social contexts. They also deploy diverse business models to serve different patient and customer needs. However, any health social enterprise can explore the gender analysis questions and findings in this study to determine the specific gender-based constraints and gender responsive strategies that resonate with their work.

Morgan et al.’s (2017) study of how gender dynamics affect maternal health and health care access in Uganda focused on the challenge of sustaining interventions that rely on external funding. At the household and community level, consistent with our findings, Morgan et al. (2017) identified economically empowering women to support themselves and their families but did not elaborate further. Our study extends Morgan et al.’s (2017) recommendation by offering a number of specific ways that female CHWs can be economically empowered, including workplace policies that promote opportunities for income generation (such as selling health products), training in financial literacy and decision-making, ensuring access to inventory supplies, and access to savings and loans to purchase inventory if needed.

Steege et al. (2020) explored why women are less represented in Mozambique’s CHW program than in other countries and identified policy recommendations designed to increase recruitment and training of female CHWs. The CHWs (known in Mozambique as APEs) are considered volunteers, although they are paid a monthly stipend equivalent to US$20. They are required to complete 4 months of away-from-home training. Four months of away-from-home training was a significant factor limiting women’s participation (due to responsibilities at home) and recommendations were made to provide childcare at training facilities and break up training into more flexible modular components that would be more accommodating to women with domestic responsibilities. The CHWs in our study are volunteers without monthly stipends although most are able to earn some extra income selling health-related products as sales agents of the social enterprises they work with. The training requirements for the CHWs we studied were 3–4 weeks which were not raised as a concern from the data we gathered. Consistent with Steege et al. (2020) however was the interest in CHWs to receive greater and more formalized compensation. In our study, greater income-generating opportunities were often provided by the social enterprises who provided inventories of health-related products and medicines that could be sold by a CHW in her community to meet local demand and generate some small amount of additional income. If greater income leads to increased empowerment for female CHWs, then exploring income-generating options pioneered by health social enterprises was a recommendation that emerged from our respondents, and a worthy avenue of further exploration.

One of the findings from our study was the need to address potential misperceptions or misunderstandings that a male spouse may have about a female CHW’s role and work responsibilities. For example, a husband or partner who was concerned that his wife was at risk of adultery by visiting community households, would not let her continue with her work. These findings are consistent with Roberton et al., [49] who explored gender differences between male and female CHWs in Tanzania and found that male and female CHW pairings could help address some of these concerns. Roberton et al., [49] also reported differences in acceptability and comfort between male and female CHWs discussing certain issues. For example, men and women reported discomfort discussing sexual and reproductive topics with members of the opposite sex. The respondents in our study identified similar concerns, and subsequently suggested that male-female CHW pairings may be a useful strategy to deal with these differences so that man-to-man and woman-to-woman discussions and counselling can occur.

### Study limitations

This study includes a number of limitations. The qualitative data gathered from CHWs and other respondents is susceptible to response bias caused by seeking social approval and acceptance. We addressed this as much as possible through lengthy focus group discussion sessions where we purposively worked to established rapport with respondents and build trust. We avoided any leading questions, kept questions short and clear, and used simple language.

Although we drew our qualitative data from women and men in Uganda and Kenya, from urban, peri-urban and rural areas, who were working with four different social enterprises with different business models, additional research could continue to explore other community health workers active in other regions of other countries working with different organizations to further test the generalizability of our findings. Since our findings were drawn from qualitative data (interviews, focus group discussions, and observations) we benefitted from understanding gender-based barriers and constraints, however, additional quantitative research with probability sampling would be able to determine how prevalent and extensive each constraint was in different contexts.

## Conclusions

The findings of this study offer practical empirical evidence that can be used by health social enterprises in Africa (as well as governments and NGOs) working with CHWs to design gender intentional strategies. The study identified seven key gender-based constraints and four interrelated pathways to integrate gender across a health social enterprise’s work with CHWs. Importantly, a health social enterprise can conduct gender analysis to better understand the influence of gender across their work and move from being a gender blind organization to a gender responsive one. There is no one tool that can address the many contexts and unique gender situations any health social enterprise must address, but the findings offer a framework to build a more gender responsive organization that supports CHWs by empowering them and increasing social benefits for themselves, their organization, their families, and their communities.

## Supplementary Information

**Additional file 1:** Annex 1. Interview and Focus Group Questions.

## Data Availability

Data gathered for the study is available from the corresponding author.

## Abbreviations

CHW: Community health worker
MCH: Maternal and child health

## Footnotes

1 Gender includes women, men, and non-binary gender, people of all genders whose “gender identity does not align with a binary understanding of gender such as man or woman. A gender identity which may include man and woman, androgynous, fluid, multiple, no gender, or a different gender outside of the ‘woman-man’ spectrum.” Status of Women Canada, (2018). Introduction to GBA+. Sex and gender – Gender diversity, page 5.

2 There are numerous terms for being gender intentional or unintentional. For our purposes, Gender Blind aligns with being Gender Exploitative, Gender Accommodating aligns with being Gender Sensitive, and Gender Responsive aligns with being Gender Transformative.

3 Domestic partner will herein be called partner. For the purpose of this study, a partner may be a husband, wife, or cohabitating partner. In addition, depending on the context and existing gender dynamics and/or the gender and sociocultural norms, another family member (e.g., mother-in-law, sister, or brother) may play an important role, especially when there is no partner or when the context dictates strong family influence.
