# Supplementary material for: Gender intentional approaches to enhance health social enterprises in Africa: a qualitative study of constraints and strategies
Source: Int J Equity Health. 2021 Apr 10;20:98. doi: 10.1186/s12939-021-01427-0 (PMC8035608; doi:10.1186/s12939-021-01427-0)
Supplement: Supplementary file 1 — Additional file 1: Annex 1. Interview and Focus Group Questions. [file 12939_2021_1427_MOESM1_ESM.docx]

**Annex 1: Interview and Focus Group Questions**

**SECTION 1: WELCOME AND INTRODUCTIONS**

**Welcome**

- Introduce the research project and why we are here today;
- Explain that this focus group is a safe space;
- Confirm that the information shared will only be used to help us learn and to improve further work with CHWs and anything they share is confidential; and
- Thank them for participating and sharing their thoughts and time with us.

**Ground Rules**

- Explain that we are looking for your honest opinions and there are no wrong answers;
- Be open and positive;
- Only one person will talk at a time; and
- Respect the opinions of everyone.

**Introductions**

- Introduce the facilitators including names, roles and where they are from; and
- Ask participants to introduce themselves including their name, age, marital status and number of children (if applicable) and how long they have been a CHW?

**SECTION 2: GETTING TO KNOW THE PARTICIPANTS**

- Why did you become a CHW? What are your primary motivations/incentives for being a CHW?
- Do you do any other activities to make money (i.e. other income generating activities)? If yes, what work/activities?

**SECTION 3: ORGANIZATION & CHWs – BENEFITS, CONSTRAINTS AND OPPORTUNITIES (EQUITABLE SYSTEMS AND STRUCTURES & GENDER RESPONSE TRAINING, SUPPORT AND INCENTIVES)**

- In thinking of your role as a CHW, what are the biggest benefits that you receive from your role as a CHW?
- In thinking of your role as a CHW, what issues and constraints do you face? **List them all down on a large flip chart.**
  - Do you face any constraints as a *female* (or male) CHW? (i.e. what constraints do you face *because* you are female or male?)
  - If applicable, do you see any differences between male and female CHW? If yes, what differences do you see?
  - If single/widowed/female head of household, do you face any additional constraints because you are single?
  - **If not mentioned as a constraint** **(Gender Responsive Training, Support and Incentives):**
    - Do you face any constraints in terms of **training**? If yes, how does this impact you?
    - Do you face any constraints in terms of **managerial/organizational support**? If yes, how does this impact you?
    - Do you face any restrictions in terms of **mobility**? If yes, how does this impact you?
    - Do you face any restrictions in terms of **finance** or access to money? If yes, how does this impact you?
    - Do you face any restrictions in terms of **decision-making** related to your business? If yes, how does this impact you?
- If there are a lot of constraints, consider prioritizing the constraints – what are the top 3 or 5 constraints that you face?
- **Working through the table below:** What opportunities do you see? Any interventions/strategies/approaches could help you to be more successful (i.e. from the organization, other, etc.)?

| **Constraint** | **Solution/Opportunity/Strategy** |
| --- | --- |
| Training |  |
| Managerial support |  |
| Mobility |  |
| Finance |  |
| Decision-making |  |
| Etc. |  |

**SECTION 4: PARTNERS (APPROPRIATE PARTNER ENGAGEMENT)**

- In thinking of your role as a CHW, does anyone help you with your role (e.g. husband/partner, kids, others, other CHWs, etc.)?
  - If yes, who and how?
  - **If partner/husband is not mentioned:**
    - Does your partner/husband play a role in your business? If yes, how?
  - Does your partner face any challenges in the support that they provide? Or do you face any challenges in how they provide support?
  - Are there ways to better support your partner’s involvement? What are your recommendations?

**SECTION 5: PATIENTS (GENDER RESPONSIVE SALES, DESIGN AND MARKETING)**

- Now, let’s think your role and job serving your community. Do your patients (i.e. mothers) face any challenges in accessing your services?
- How could the services or products you offer meet the needs of your patients (i.e. mothers) better?
  - **If not mentioned as a constraint – consider asking the following:**
    - Do you face any constraints in terms of providing **family planning services**? Do your patients face any constraints in terms of accessing family planning services? If yes, how?
    - Do you face any constraints in terms of addressing **gender-based violence services**? Do your patients face any constraints in terms of accessing gender-based violence services? If yes, how?
  - **Working through the table below:** What opportunities do you see? Any interventions/strategies/approaches could help you to be more successful (i.e. from the organization, other, etc.)?

| **Constraint** | **Solution/Opportunity/Strategy** |
| --- | --- |
|  |  |
|  |  |

**SECTION 6: CLOSING**

- Do you have any questions for us?
- Is there anything else that you would like to share or anything else that you would like to discuss?
- In closing, I would like to reiterate how helpful you have been. Thank you for your help and for sharing your time.
